# Moderate elevation of serum uric acid levels improves short-term functional outcomes of ischemic stroke in patients with type 2 diabetes mellitus

**DOI:** 10.1186/s12877-023-04141-4

**Published:** 2023-07-19

**Authors:** Yalun Dai, Yingyu Jiang, Luping Zhang, Xin Qiu, Hongqiu Gu, Yong Jiang, Xia Meng, Zixiao Li, Yongjun Wang

**Affiliations:** 1 Department of Neurology, Beijing Tiantan Hospital, Capital Medical University, Beijing, China; 2 China National Clinical Research Center for Neurological Diseases, Beijing Tiantan Hospital, Capital Medical University, No.119 South 4th Ring West Road, Fengtai District, Beijing, 100070 China; 3 Department of Obstetrics and Gynecology, Beijing Tiantan Hospital, Capital Medical University, Beijing, China; 4 Chinese Institute for Brain Research, Beijing, China

**Keywords:** Serum uric acid, Ischemic stroke, Diabetes mellitus, Functional outcome, Prognosis

## Abstract

**Background:**

Serum uric acid (SUA), an end-product of purine catabolism diffused in the blood, is positively associated with the risk of type 2 diabetes mellitus (T2DM). However, in the T2DM population, the association of SUA fluctuation ($\Delta$SUA) with the functional outcome of ischemic stroke (IS) is still unclear. Accordingly, this study aimed to assess the correlation between $\Delta$SUA and short-term IS functional outcomes in T2DM patients.

**Methods:**

All T2DM patients diagnosed with IS in the China National Stroke Registry III were included. $\Delta$SUA, which was defined as the difference between the SUA levels at baseline and 3 months after symptom onset, was classified into two groups, i.e., elevated $\Delta$SUA ($\Delta$SUA > 0) and reduced $\Delta$SUA ($\Delta$SUA $\le$ 0). The outcomes measured using the Modified Rankin Scale (mRS) were scored from 0 to 6, and poor functional outcome was defined as an mRS score of 3–6 at 3 months after IS.

**Results:**

Among the 1255 participants (mean age: 61.6 ± 9.8 years), 64.9% were men. Patients with elevated $\Delta$SUA had a lower incidence of poor functional outcomes at 3 months. Compared with reduced $\Delta$SUA, elevated $\Delta$SUA at 0–50 μmol/L (odds ratio [OR] = 0.46, 95% confidence interval [CI] = 0.28–0.78, *p* = 0.004) and 50–100 μmol/L (OR = 0.40, 95% CI = 0.21–0.77, *p* = 0.006) was significantly correlated with a reduced risk of poor functional outcomes at 3 months.

**Conclusion:**

This study showed that a moderate increase in $\Delta$SUA in the range of 0–100 μmol/L at 3 months after IS might be beneficial in T2DM adults and more studies are warranted to confirm this.

**Supplementary Information:**

The online version contains supplementary material available at 10.1186/s12877-023-04141-4.

## Background

Stroke is a leading cause of disability and death globally [1], and ischemic stroke (IS), one of the major pathological types of stroke, accounts for 62.4% of all new cases of stroke [2]. Uric acid is an end-product of purine catabolism and is involved in endogenous and exogenous oxidative stress processes [3]. Serum uric acid (SUA) levels are influenced by the net balance of reabsorption and secretion via the intestines and kidneys [4]. SUA is considered to be a biomarker of metabolic disturbance [5]. Converging evidence has demonstrated that maintaining moderate SUA levels is associated with a decreased risk of cerebral hypometabolism and cognitive impairment [6, 7]. SUA has attracted considerable attention because SUA levels are relevant to the functional outcomes of IS. Several studies have demonstrated evidence linking higher SUA levels with increased IS incidence and mortality [8]; however, others have reported that SUA has a protective effect on IS prognosis [9]. Moreover, lower SUA levels have been strongly associated with short-term poor prognosis in IS [10]. Interestingly, a U-shaped model was recently presented to clarify the association between SUA levels and IS prognosis [11].

Accumulating evidence indicates that high SUA levels are related to the prevalence of type 2 diabetes mellitus (T2DM) as a consequence of their strong correlation with a pro-oxidative and pro-inflammatory state [3, 12]. Researchers have reported that high SUA levels worsen IS prognosis in the T2DM population [13]. SUA levels are sensitive and fluctuate with diet, lifestyle, and the presence of cardio/cerebrovascular and metabolic diseases [3], meaning most changes are usually subtle. However, whether SUA fluctuation ($\Delta$SUA) has an important influence on IS prognosis in T2DM patients remains uncertain. In addition, only considering the SUA levels makes it difficult to appropriately reflect the metabolic homeostasis of IS in T2DM patients. Hence, the objective of this study was to evaluate the effect of $\Delta$SUA upon the short-term IS prognosis in T2DM patients.

## Methods

### Study population

The Third China National Stroke Registry (CNSR-III) was a large multi-center prospective cohort study conducted from 2015 to 2018, in which a total of 15,166 participants diagnosed with IS or transient ischemic attack (TIA) were enrolled from 201 registries and hospitals in China. TIA is an episode of focal neurological dysfunction lasting less than 24 h, which usually manifests as a symptom complex lasting only minutes with no cerebral infarction. Neurological deficit symptoms lasting more than 24 h with cerebral infarction were defined as IS. The diagnosis of TIA or IS is confrimed by brain imaging in the CNSR-III [14]. Individuals had silent cerebral infarction without symptom manifestation or those who refused to participate in the cohort study were excluded. T2DM was diagnosed as a non-insulin diabetes mellitus at discharge, adding that having a fasting plasma glucose (FPG) level of ≥ 7.0 mmol/L (126 mg/dL) or hemoglobin A1c (HbA1c) level of ≥ 6.5%. Participants over 18 years of age were eligible for the study and were enrolled within 7 days after symptom onset. The specific description and protocol of the CNSR-III have been published previously [14]. This study included 1255 T2DM individuals diagnosed with IS who had complete SUA data. The CNSR-III was conducted in accordance with the Helsinki Declaration and approved by the ethics committees of the Beijing Tiantan Hospital (No. KY2015-001–13) and other branch centers. Each patient provided written informed consent prior to participation in the study.

### Data collection and assessment of $\Delta$SUA

In the CNSR-III, baseline clinical data were collected through direct interviews by trained research coordinators and from medical records at each site. It included age, sex, body mass index (BMI; calculated as weight [kg]/square of height [m^2^]), heavy drinking and current smoking status; diastolic and systolic blood pressure at admission; medical histories of hyperlipidemia, hypertension, and thrombolytic therapy; and National Institutes of Health Stroke Scale (NIHSS) scores at admission and discharge. Every fasting blood sample was gathered within 24 h of admission and at 3 months, as well as stored at each clinical site. All blood samples were frozen in cryotubes at -80 $^\circ{\rm C}$ and conveyed to the central laboratory via a cold chain system. Laboratory tests were performed centrally to obtain relevant plasma parameters, including HbA1c, FPG, high-density lipoprotein cholesterol (HDL), total cholesterol (CHOL), low-density lipoprotein cholesterol (LDL), and triglyceride (TG) levels at baseline, as well as SUA and high-sensitivity C-reactive protein (hsCRP) at baseline and 3 months after symptom onset.

$\Delta$SUA was calculated as the value of the SUA level at 3 months after symtom onset minus that at baseline. Further, participants were classified into two groups based on ∆SUA as follows: elevated ($\Delta$SUA > 0) and reduced ($\Delta$SUA $\le$ 0) $\Delta$SUA groups.

### Assessment of outcomes

Each patient was followed up with an in-person interview for clinical outcomes at 3 months after symptom onset. The functional outcome was determined using the modified Rankin scale (mRS) at 3 months [15, 16]. The mRS scores range from 0–6, with 3–6 being defined as poor functional outcomes and 0–2 as favorable functional outcomes. Every event was recorded after a double-blind comprehensive assessment.

### Definitions of other variables

Smoking status was categorized as “current smoking” or not. “Current smoking” was defined as active smoking at the time of IS and non-current smoking as “smoked previously” or “never smoked.” Active smoking refers to smokers with a habit of smoking rather than passively inhaling second-hand smoke. Alcohol consumption was classified as “heavy drinking” or not. “Heavy drinking” was defined as consistently consuming $\ge$ 20 g/day of standard-size alcoholic beverages, and non-heavy drinking was categorized as “moderate,” “mild,” or “never” drinking. The difference between the hsCRP levels at baseline and 3 months after onset was defined as hsCRP fluctuation ($\Delta$ hsCRP), and $\Delta$hsCRP increase and decrease were presented as $\Delta$hsCRP > 0 and $\Delta$hsCRP $\le$ 0, respectively. On the basis of the Trail of ORG 10172 in Acute Stroke Treatment (TOAST) criteria [17], IS subtypes were classified as large artery atherosclerosis (LAA), cardioembolism (CE), small artery occlusion (SAO), as well as other determined and undetermined causes. In this study, other determined or undetermined causes were defined as “others” [18].

### Statistical analyses

Continuous variables conforming to a normal distribution are presented as mean ± standard deviation (SD) with a t-test or one-way analysis of variance (ANOVA), and median (interquartile range, IQR) with the Kruskal–Wallis test as appropriate. Categorical variables are expressed as frequencies and percentages, as well as were analyzed with a chi-squared ($\chi$^2^) test among multiple groups. Binary logistic regression was chosen to analyze the correlation between $\Delta$SUA and poor functional outcome at 3 months with the following three models: unadjusted, model 1, and model 2. The reduced ∆SUA group was selected as the reference group in accordance with reduced ∆SUA being correlated with short-term poor functional outcomes after IS [10]. Model 1 was adjusted for age, sex, BMI, NIHSS at admission, current smoking status, and heavy alcohol consumption. Model 2 was further adjusted for medical history of heart disease, hypertension, hyperlipidemia, and thrombolytic therapy, as well as serum lipid parameters (CHOL, TG, and LDL). In addition, the interaction of age, sex, inflammatory biomarkers, and TOAST classification was assessed through a subgroup analysis adjusted for the variables in model 2. Then, considering the potential effects of blood glucose parameters on outcomes, a sensitivity analysis was performed by further adjusting for HbA1c and FPG respectively in model 2. The strength of the associations was assessed by odds ratios (ORs) with 95% confidence intervals (CIs). SAS software version 9.4 (SAS Institute, Inc., Cary, North Carolina) was used for all statistical analyses. A *p*-value of < 0.05 (two-sided) was considered statistically significant.

## Results

### Baseline characteristics of participants

**Fig. 1:**
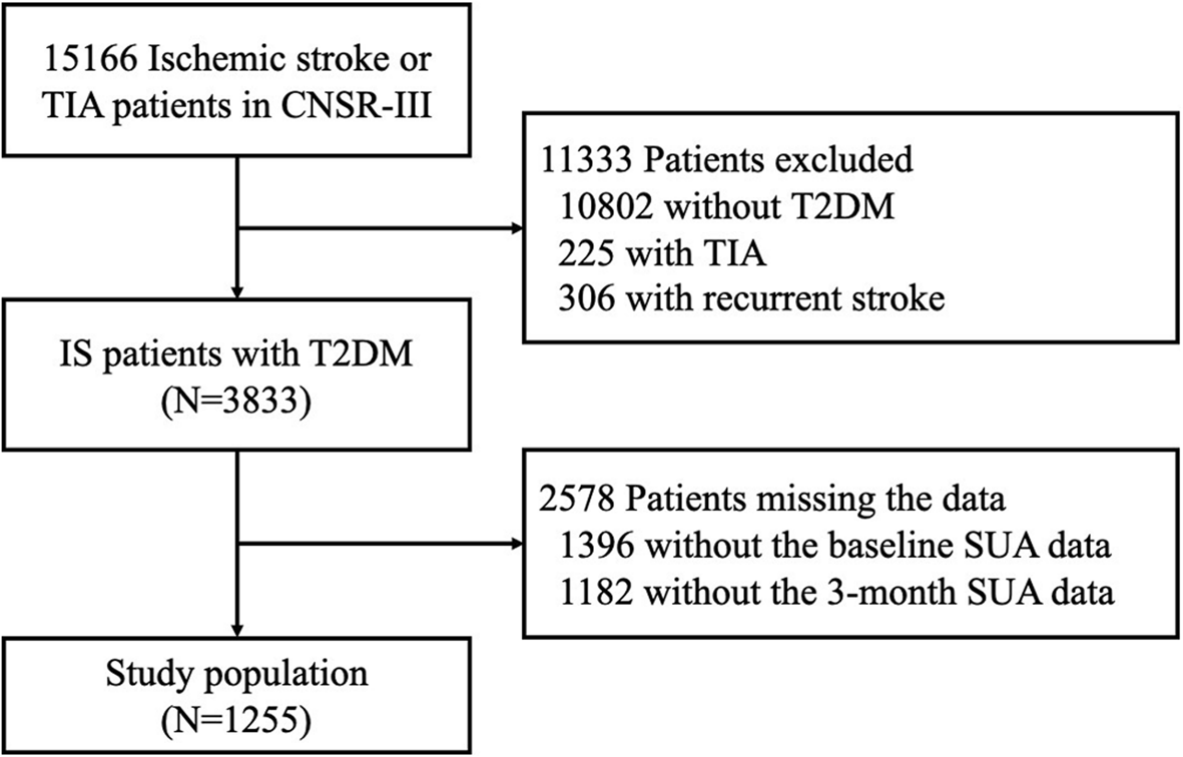
Flowchart showing the selection process of patients included in this study. Abbreviations: TIA, transient ischemic attack; CNSR-III, China National Stroke Registry III; T2DM, type 2 diabetes mellitus; SUA, serum uric acid

From the 15,166 participants in the CNSR-III, we excluded 10,802 non-diabetes patients, 225 TIA patients, 306 recurrent stroke patients, 1396 patients without baseline SUA data, and 1182 patients without 3-month SUA data. Finally, a total of 1255 patients were eligible for inclusion in the present study (Fig. 1). Among the 1255 patients (mean age, 61.6 ± 9.8 years), 815 (64.9%) were male. A summary of baseline characteristics in the elevated and reduced $\Delta$SUA groups is shown in Table 1. In the elevated $\Delta$SUA group, the proportions of patients that smoked and patients with thrombolytic therapy were higher compared to those in the reduced $\Delta$SUA group. Baseline SUA in the elevated $\Delta$SUA group was lower than that in the reduced $\Delta$SUA group (*p* < 0.001), and the opposite result was obtained for 3-month SUA (*p* < 0.001). In the population with HbA1c or FPG data, HbA1c and FPG levels were both higher in the elevated $\Delta$SUA group than in the reduced $\Delta$SUA group (HbA1c: *p* = 0.020; FPG: *p* = 0.007). Regarding blood biomarkers, patients with elevated ∆SUA had lower mean levels of hsCRP (3.6 ± 10.8 vs. 6.1 ± 25.4, *p* = 0.037), CHOL (4.1 ± 1.1 vs. 4.4 ± 1.3, *p* < 0.001), TG (1.7 ± 0.9 vs. 1.9 ± 1.3, *p* = 0.002), and LDL (2.3 ± 1.0 vs. 2.6 ± 1.1, *p* < 0.001) at 3 months than those with reduced ∆SUA. In addition, $\Delta$hsCRP in the elevated ∆SUA group was 7.5 mg/L lower than that in the reduced ∆SUA group (3.5 ± 12.0 vs. 10.6 ± 39.7, *p* = 0.018).

**Table 1:** Clinical characteristics of IS patients with T2DM stratified by ∆SUA

**Variables**	**Total (*N* = 1255 [100%])**	$\Delta$**SUA > 0 (*N* = 764 [60.9%])**	$\Delta$**SUA** $\le$ **0 (*N* = 491 [39.1%])**	***P* value**
Demographic characteristics				
Age, years, mean ± SD	61.6 ± 9.8	61.8 ± 10.1	62.4 ± 10.5	0.317
Sex, male, N (%)	815 (64.9)	503 (65.8)	312 (63.5)	0.406
BMI (kg/m^2^), mean ± SD	25.3 ± 3.3	25.2 ± 3.3	25.4 ± 3.3	0.419
Current smoking, N (%)	368 (29.3)	247 (32.3)	121 (24.6)	**0.004**
Heavy drinking, N (%)	154 (12.3)	103 (13.5)	51 (10.4)	0.103
Medical history, N (%)				
Heart disease	182 (14.5)	110 (14.4)	72 (14.7)	0.896
Hypertension	980 (78.1)	593 (77.6)	387 (78.8)	0.616
Hyperlipidemia	151 (12.0)	98 (7.8)	53 (4.2)	0.280
Thrombolytic therapy	100 (8.0)	75 (9.8)	25 (5.1)	**0.003**
Laboratory test, mean ± SD				
SBP at admission (mmHg)	152.7 ± 21.6	152.5 ± 21.5	153.0 ± 21.6	0.687
DBP at admission (mmHg)	87.3 ± 12.7	86.9 ± 12.5	87.8 ± 12.9	0.263
NIHSS at admission	4.1 ± 3.4	4.1 ± 3.6	4.1 ± 3.2	0.784
NIHSS at discharge	2.3 ± 2.6	2.3 ± 2.5	2.4 ± 2.7	0.412
Baseline hsCRP (mg/L)	6.1 ± 21.8	6.3 ± 21.2	5.7 ± 22.7	0.606
> 1.8	11.2 ± 30.0	11.9 ± 29.2	10.3 ± 31.2	0.507
≤ 1.8	0.9 ± 0.4	0.9 ± 0.4	0.9 ± 0.4	0.509
3-month hsCRP (mg/L)	4.6 ± 18.0	3.6 ± 10.8	6.1 ± 25.4	**0.037**
> 1.3	8.5 ± 25.0	6.7 ± 15.0	11.0 ± 34.3	0.059
≤ 1.3	0.8 ± 0.3	0.8 ± 0.3	0.7 ± 0.3	0.348
$\Delta$ hsCRP (mg/L)	-1.5 ± 27.0	-2.7 ± 21.4	0.5 ± 33.8	0.064
> 0	6.1 ± 26.2	3.5 ± 12.0	10.6 ± 39.7	**0.018**
≤ 0	-6.6 ± 26.2	-7.1 ± 25.2	-5.9 ± 27.8	0.516
HbA1c (%)^a^	8.1 ± 2.0	8.3 ± 2.0	7.9 ± 1.9	**0.020**
FPG (mmol/L)^a^	9.1 ± 3.4	9.3 ± 3.5	8.7 ± 3.2	**0.007**
CHOL (mmol/L)	4.2 ± 1.2	4.1 ± 1.1	4.4 ± 1.3	** < 0.001**
TG (mmol/L)	1.8 ± 1.0	1.7 ± 0.9	1.9 ± 1.3	**0.002**
HDL (mmol/L)	0.9 ± 0.3	0.9 ± 0.3	0.9 ± 0.3	0.560
LDL (mmol/L)	2.4 ± 1.0	2.3 ± 1.0	2.6 ± 1.1	** < 0.001**
Baseline SUA, mean ± SD	294.1 ± 90.1	268.8 ± 79.3	334.0 ± 91.5	** < 0.001**
Baseline SUA, median (IQR)	287.0 (231.0–347.0)	261.0 (213.5–314.0)	330.0 (272.0–383.0)	** < 0.001**
3-month SUA, mean ± SD	315.1 ± 93.2	337.4 ± 94.7	280.5 ± 79.3	** < 0.001**
3-month SUA, median (IQR)	307.0 (252.0–365.0)	325.0 (272.0–389.0)	277.0 (230–327.0)	** < 0.001**
Functional outcome				
3-month mRS 3–6, N (%)	139 (11.1)	69 (9.0)	70 (14.3)	**0.004**

Continuous data are presented as mean (standard deviation, SD) and median (interquartile range, IQR), and categorical variables are presented as %

*IS* Ischemic stroke, *T2DM* Type 2 diabetes mellitus, $\Delta$*SUA* Changes in serum uric acid, *BMI* Body mass index, *SBP* Systolic blood pressure, *DBP* Diastolic blood pressure, *NIHSS* National Institutes of Health Stroke Scale, *hsCRP* Hypersensitive C-reactive protein, $\Delta$*hsCRP* Difference between hypersensitive C-reactive protein values at baseline and at 3 months after symptom onset, *IL6* Interleukin 6, *HbA1c* Hemoglobin A1c, *FPG* Fasting plasma glucose, *CHOL* Total cholesterol, *TG* Triglyceride, *HDL* High-density lipoprotein, *LDL* Low-density lipoprotein

^a^Population with missing data: 382 (30.4%) and 277 (22.1%) lacked HbA1c and FPG data, respectively

### Association between $\Delta$SUA and poor functional outcomes

**Fig. 2:**
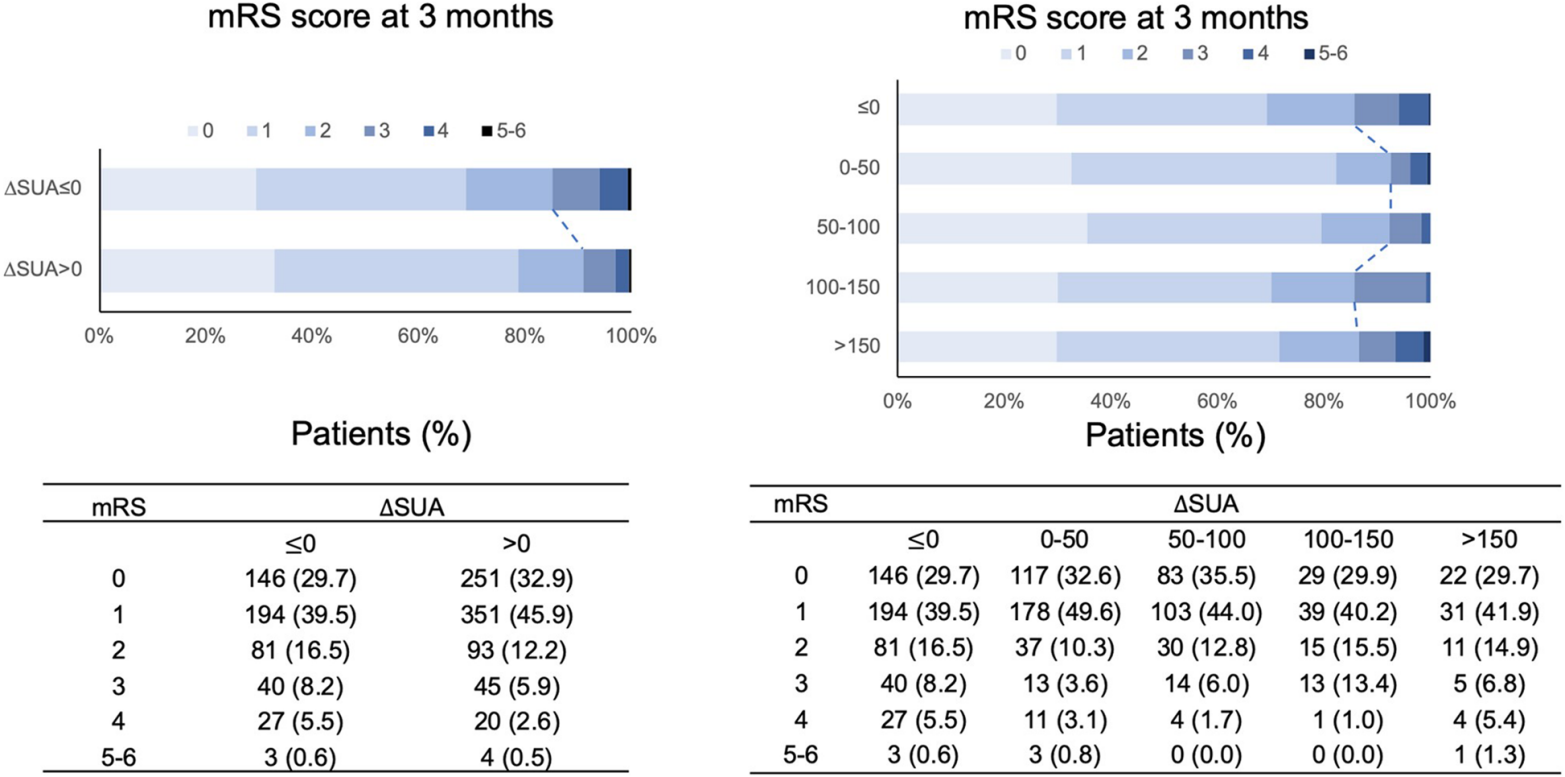
Distribution of modified Rankin Scale (mRS) scores* stratified by $\Delta$SUA. *mRS scores ranged from 0 to 6, with 0 indicating no symptoms; 1, no clinical disability; 2, slight disability; 3, moderate disability; 4, moderately severe disability; 5, severe disability; and 6, death. $\Delta$SUA, changes in serum uric acid; mRS, modified Rankin Scale

During the 3-month follow-up, 11.1% (139/1255) of patients had a poor functional outcome (Table 1). Patients with elevated $\Delta$SUA had a lower risk of poor functional outcomes than those with reduced $\Delta$SUA (9.0% vs. 14.3%, *p* = 0.004). In particular, the risk of poor outcome was 7.5% and 7.7% in patients within 0–50 μmol/L and 50–100 μmol/L elevated $\Delta$SUA, respectively. The distribution of ∆SUA and the functional outcomes of IS are presented in Fig. 2.

The association of $\Delta$SUA with poor functional outcomes is shown in Table 2. In the unadjusted logistic regression model, elevated ∆SUA was significantly correlated with a better prognosis of IS compared with reduced $\Delta$SUA. We further categorized the elevated ∆SUA group into four subgroups (i.e., 0–50 μmol/L, 50–100 μmol/L, 100–150 μmol/L, and > 150 μmol/L), where a multi-classification analysis revealed that the 0–50 μmol/L (OR = 0.49, 95% CI = 0.31–0.78, *p* = 0.003) and 50–100 μmol/L (OR = 0.50, 95% CI = 0.29–0.86, *p* = 0.013) $\Delta$SUA subgroups had a statistically significant association with favorable functional outcomes. In model 1, when adjustments for sociodemographic characteristics were made, the association was slightly enhanced and stabilized in the 0–50 μmol/L (OR = 0.46, 95% CI = 0.28–0.77, *p* = 0.003) and 50–100 μmol/L (OR = 0.40, 95% CI = 0.21–0.77, *p* = 0.006) subgroups. In model 2, after further adjustments for a history of diseases and several serum lipid parameters, similar results were observed in the 0–50 μmol/L (OR = 0.46, 95% CI = 0.28–0.78, *p* = 0.004) and 50–100 μmol/L (OR = 0.40, 95% CI = 0.21–0.76, *p* = 0.006) subgroups.

**Table 2:** Binary/multivariate logistic regression analyses of the association of $\Delta$SUA with poor functional outcomes of IS

$\Delta$**SUA value (μmol/L)**	**No. of event/No. at risk**	**Unadjusted**		**Model 1**^a^		**Model 2**^b^	
		OR (95% CI)	*P* value	OR (95% CI)	*P* value	OR (95% CI)	*P* value
$\Delta$SUA $\le$ 0	70/491	reference	-	reference	-	reference	-
$\Delta$SUA > 0	69/764	0.60 (0.42–0.85)	**0.004**	0.54 (0.36–0.80)	**0.002**	0.53 (0.35–0.80)	**0.002**
0–50	27/359	0.49 (0.31–0.78)	**0.003**	0.46 (0.28–0.77)	**0.003**	0.46 (0.28–0.78)	**0.004**
50–100	18/234	0.50 (0.29–0.86)	**0.013**	0.40 (0.21–0.77)	**0.006**	0.40 (0.21–0.77)	**0.006**
100–150	14/97	1.01 (0.55–1.89)	0.964	1.06 (0.53–2.12)	0.877	1.03 (0.51–2.11)	0.931
> 150	10/74	0.94 (0.46–1.92)	0.864	0.72 (0.30–1.72)	0.462	0.72 (0.30–1.73)	0.459

$\Delta$*SUA* Changes in serum uric acid, *IS* Ischemic stroke, *OR* Odds ratio, *CI* Confidence interval, *BMI* Body mass index, *NIHSS* National Institutes of Health Stroke Scale, *CHOL* Total cholesterol, *TG* Triglyceride, *LDL* Low-density lipoprotein

^a^Model 1 adjusted for age, sex, BMI, NIHSS at admission, current smoking, and heavy alcohol consumption

^b^Model 2 adjusted for age, sex, BMI, NIHSS at admission, current smoking, heavy alcohol consumption, history of disease (i.e., heart disease, hypertension, and hyperlipidemia), CHOL, TG, and LDL

### Sensitivity analyses

HbA1c and FPG are critical blood glucose parameters for the diagnosis of T2DM and were further adjusted for in model 2 (Additional file 1). Similar result that elevated $\Delta$SUA was associated with better functional outcomes of IS in the range of 0–100 μmol/L was observed for both HbA1c and FPG in binary models (*p* < 0.05); although the *p*-values approached the threshold in the multivariate regression models.

### Subgroup analyses

**Fig. 3:**
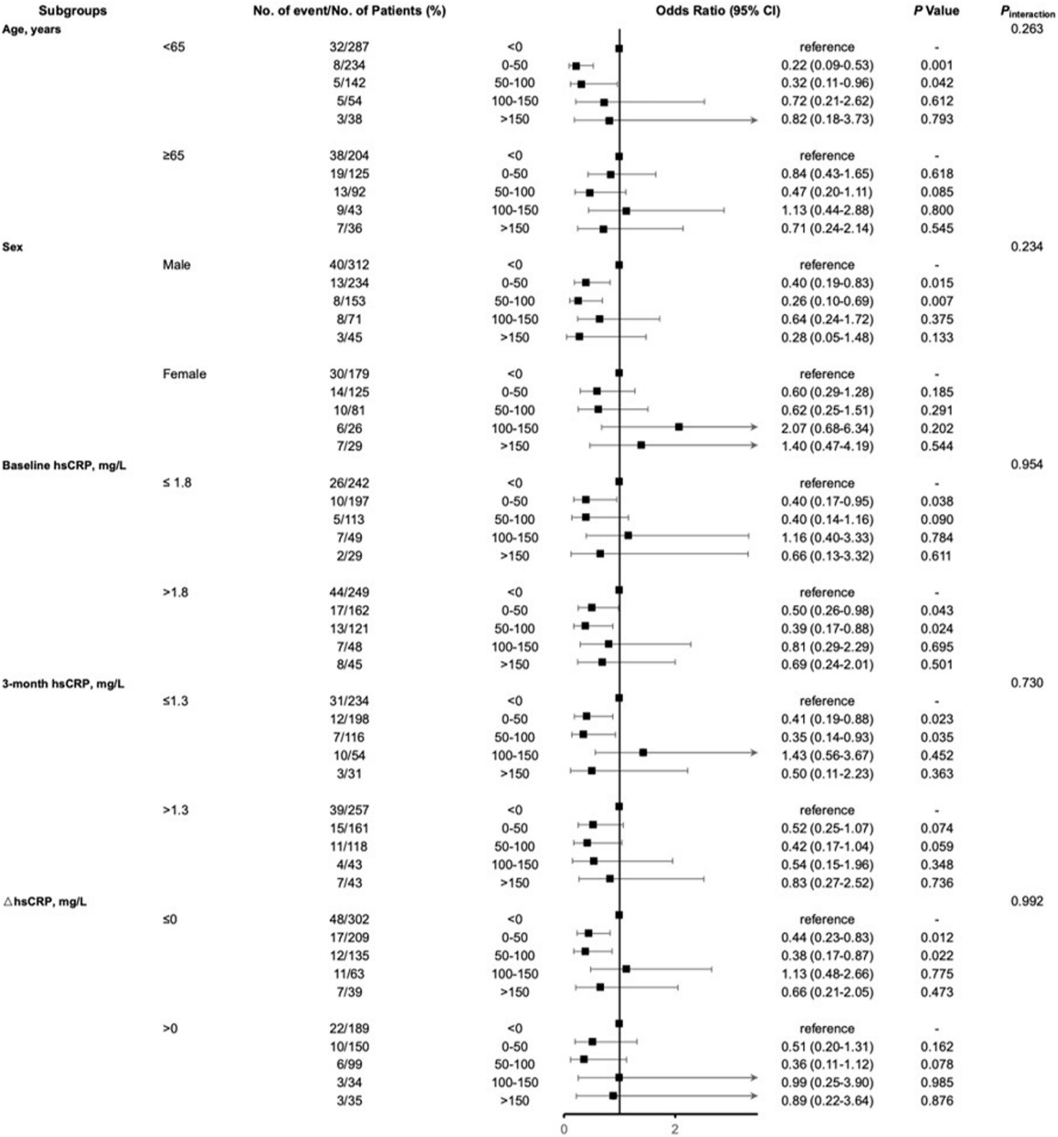
Subgroup analysis* of the association between elevated ∆SUA and poor functional outcomes. *Subgroup analysis was adjusted for age, sex, BMI, NIHSS at admission, current smoking status, heavy alcohol consumption, disease history (i.e., heart disease, hypertension, and hyperlipidemia), CHOL, TG, and LDL. $\Delta$SUA, changes in serum uric acid; OR, odds ratio; CI, confidence interval; hsCRP, high-sensitivity C-reactive protein; $\Delta$hsCRP, changes in high-sensitivity C-reactive protein; BMI, body mass index; NIHSS, National Institutes of Health Stroke Scale; CHOL, total cholesterol; TG, triglyceride; LDL, low-density lipoprotein

In patients with elevated $\Delta$SUA, we further analyzed subgroups stratified by age, sex, and several inflammatory biomarkers (Fig. 3). Patients were grouped into under 65 and over 65 years of age based on the definition of older adults by the World Health Organization. Baseline hsCRP, 3-month hsCRP, and $\Delta$hsCRP were each classified into two subgroups according to their median values in the study population. Being under 65 years old and male was significantly correlated with a favorable functional outcome in the 0–50 μmol/L (under 65 years old: OR = 0.22, 95% CI = 0.09–0.53, *p* = 0.001; being male: OR = 0.40, 95% CI = 0.19–0.83, p = 0.015) and 50–100 μmol/L (under 65 years old: OR = 0.32, 95% CI = 0.11–0.96, p = 0.042; being male: OR = 0.26, 95% CI = 0.10–0.69,* p* = 0.007) elevated $\Delta$SUA subgroups. Regarding inflammatory biomarkers, > 1.8 mg/L of baseline hsCRP (0–50 μmol/L subgroup: OR = 0.50, 95% CI = 0.26–0.98, *p* = 0.043; 50–100 μmol/L subgroup: OR = 0.39, 95% CI = 0.17–0.88, *p* = 0.024), < 1.3 mg/L of 3-month hsCRP (0–50 μmol/L subgroup: OR = 0.41, 95% CI = 0.19–0.88, *p* = 0.023; 50–100 μmol/L subgroup: OR = 0.35, 95% CI = 0.14–0.93, *p* = 0.035), and < 0 mg/L $\Delta$hsCRP (0–50 μmol/L subgroup: OR = 0.44, 95% CI = 0.23–0.83,* p* = 0.012; 50–100 μmol/L subgroup: OR = 0.38, 95% CI = 0.17–0.87, *p* = 0.022) were significantly correlated with better functional outcomes of IS in the 0–50 μmol/L and 50–100 μmol/L elevated $\Delta$SUA subgroups. However, the interaction between age, sex, inflammatory biomarkers, and $\Delta$SUA for the risk of functional outcomes of IS was not significant (all *p* > 0.05).

The results after the TOAST stratification are shown in Additional file 2. In the LAA group, $\Delta$SUA elevation was positively associated with better functional outcomes (OR = 0.29, 95% CI = 0.13–0.63, *p* = 0.002), especially in the 0–50 μmol/L (OR = 0.24, 95% CI = 0.08–0.70, *p* = 0.009) and 50–100 μmol/L (OR = 0.27, 95% CI = 0.08–0.85, *p* = 0.025) elevated $\Delta$SUA subgroups. No such trend was observed in other IS subtype groups. In addition, the interactions between TOAST classification and $\Delta$SUA for the risk of functional outcomes of IS were not significant in binary or multivariable models (both *p* > 0.05).

In elevated $\Delta$SUA subgroups, we also presented the mean ± SD and median (IQR) values of SUA levels at baseline and 3 months after discharge (Additional file 3). Baseline SUA was well balanced (*p* = 0.067) in the four subgroups (i.e., 0–50 μmol/L, 50–100 μmol/L, 100–150 μmol/L, and > 150 μmol/L), while 3-month SUA rised gradually with the increasing level of subgroups (*p* < 0.001); median levels also had an increasing trend (*p* < 0.001).

## Discussion

The main finding of this cohort study is that elevated $\Delta$SUA at 3 months in the range of 0–100 μmol/L was associated with a reduced risk of poor functional outcomes of IS in T2DM patients compared to reduced $\Delta$SUA. However, beyond this range, the association of elevated ∆SUA with functional outcomes of IS was not statistically significant.

$\Delta$SUA is an emerging indicator that has recently been used to evaluate the risk of stroke [19, 20] and T2DM [21]. Accumulating studies indicate that SUA levels [22, 23] and hyperuricemia [24] are common metabolic indicators that can be used to assess the IS prognosis in patients with pathoglycemia. For example, Wang et al. [22] reported that higher SUA levels were independently positively associated with the risk of IS in T2DM population, and in another study having smaller samples, the result revealed that an increased SUA level was correlated with higher risks of mortality and recurrent vascular events [23]. In addition, hyperuricemia, as a threshold for high SUA levels, was an independent prognostic factor for poor in-hospital outcomes of IS [24]. However, neither of the indicators mentioned above has adequate sensitivity or specificity to identify the contribution of SUA to IS prognosis in T2DM patients. Notably, a prospective cohort study that included 51,441 participants [19] suggested that elevated $\Delta$SUA might be linked to the incidence of hemorrhagic stroke but not IS. Moreover, a 7-year cohort study reported that elevated $\Delta$SUA over time would raise the risk for all types of strokes, and there was no safe dose of elevated $\Delta$SUA [20]. Similar results were found for elevated $\Delta$SUA in the T2DM population [21]. However, more evidence is needed to determine whether $\Delta$SUA has a significant effect on IS prognosis in T2DM patients. Considering that $\Delta$SUA can be affected by multiple factors, this study focused on investigating the relationship between ∆SUA and short-term prognosis of IS after adjusting for sociodemographic factors, medical histories, and several lipid parameters. Our results were inconsistent with findings in previous studies. Still, they demonstrated that elevated ∆SUA within a certain range (0–100 μmol/L) might have a protective effect on the functional outcomes of IS compared to reduced ∆SUA, especially in LAA subtypes of IS. However, elevated ∆SUA beyond such a range had no beneficial effect. Even though the data of elevated $\Delta$SUA higher than 100 μmol/L were insufficient, it is reasonable to assume that there might have a U-shaped correlation between elevated $\Delta$SUA and functional outcomes of IS. Such a non-linear relationship would be somewhat consistent with findings in previous studies on SUA levels [11, 22].

In the subgroup analysis, we found that changes in hsCRP at 3 months after IS might have a distinct effect on the correlation between elevated $\Delta$SUA and short-term functional outcomes. In the subgroup with a reduction in hsCRP, moderately elevated $\Delta$SUA (range: 0–100 μmol/L) tended to decrease the risk of poor functional outcomes of IS. However, how $\Delta$SUA and $\Delta$hsCRP interact with each other remains unclear. One retrospective study demonstrated that elevated hsCRP was positively associated with high SUA level in a healthy Chinese population [25], whereas another clinical study of T2DM found no synergistic interaction between SUA and hsCRP [26]. The present study provides evidence of a positive role for modestly elevated $\Delta$SUA in favorable functional outcomes of IS in T2DM patients with hsCRP reduction. These findings suggest that high SUA levels in patients with metabolic diseases (such as T2DM), unlike in healthy people, might not exert a negative effect on inflammation. Further studies are warranted to identify the correlation between $\Delta$SUA and $\Delta$hsCRP at the IS patients with T2DM.

The antioxidant effects of SUA might underlie the relationship between $\Delta$SUA and the short-term functional outcomes of IS. A moderate concentration of SUA could protect tissues and organs against oxidative damage to maintain metabolism balance [27, 28]. SUA is generally scarce in the human brain, which makes it more susceptible to oxidative stress [29]. For IS patients, low SUA levels, when maintained for up to 3 months after symptom onset, tended to be positively associated with poor functional outcomes according to the URICO-ICTUS trial [30]. In conclusion, our finding that elevated $\Delta$SUA is associated with favorable functional outcomes of IS in the range of 0–100 μmol/L is in line with the aforementioned evidence, despite a J-shaped risk trend for the incidence of IS reported in other studies [8]. Notably, recent evidence has shown that SUA intervention might improve glucose-driven oxidative stress in IS, providing a neuroprotective effect in hyperglycemia patients [31]. Large-scale studies are warranted to further explore the effect of moderately elevated $\Delta$SUA on the functional outcomes of IS in T2DM patients.

Moreover, the SUA level and its changes may play a role in metabolism-related diseases and, thus, influence the clinical prognosis. Persistent low SUA was associated with Alzheimer-related cerebral hypometabolism in a prospective cohort study on Alzheimer’s disease [6]. In addition, the Chinese Health and Retirement Longitudinal Study revealed that maintaining a higher SUA level within the normal range and a moderate increase in $\Delta$SUA could improve cognitive function in women with high FPG and non-normotensive population, respectively [32, 33]. Therefore, a moderate increase in $\Delta$SUA might be advantageous at cerebral function in individuals with metabolism-related diseases, although further longitudinal studies are needed to confirm this.

The strength of this study is that we analyzed and explored the relationship of dynamic changes in serum indicators ($\Delta$SUA) with IS in a multicenter prospective registry. However, this study also has some limitations. First, all patients were Chinese, so the present finding needs to be replicated in populations of different ethnicities and ancestries. Second, since the dynamic changes of other inflammatory biomarkers were not monitored, the potential applications of the inflammatory mechanisms are limited. Third, the sample size in this study was not large enough to evaluate other outcomes, such as IS recurrence or death.

## Conclusion

This study revealed that moderately elevated $\Delta$SUA was correlated with better short-term functional outcomes in IS patients with T2DM and might have a more pronounced protective effect in the reduced $\Delta$hsCRP subgroup. These findings suggest that $\Delta$SUA might have a subtle metabolic correlation with IS in T2DM patients.

### Supplementary Information

**Additional file 1.** Binary/multivariate logistic regression analyses of the association of $\Delta$SUA with poor functional outcomes of IS in the population with HbA1c or FPG, table.

**Additional file 2.** Subgroup analysis of the association between $\Delta$SUA and poor functional outcomes of IS, table.

**Additional file 3.** Distribution of SUA levels in $\Delta$SUA elevation subgroups, figure.

## Data Availability

The dataset supporting the conclusions of this article are available from the corresponding author upon reasonable request.

## Abbreviations

BMI: Body mass index
CE: Cardioembolism
CHOL: Total cholesterol
CI: Confidence interval
CNSR-III: Third China National Stroke Registry
FPG: Fasting plasma glucose
HbA1c: Hemoglobin A1c
HDL: High-density lipoprotein cholesterol
hsCRP: High-sensitivity C-reactive protein
IQR: Interquartile range
IS: Ischemic stroke
LAA: Large-artery atherosclerosis
LDL: Low-density lipoprotein cholesterol
mRS: Modified Rankin Scale
NIHSS: National Institutes of Health Stroke Scale
OR: Odds ratio
SAO: Small artery occlusion
SD: Standard deviation
SUA: Serum uric acid
T2DM: Type 2 diabetes mellitus
TG: Triglyceride
TIA: Transient ischemic attack
TOAST: Trial of ORG 10172 in Acute Stroke Treatment

## References

[1] Tian DS, Liu CC, Wang CL, Qin C, Wang MH, Liu WH, et al. Prevalence and risk factors of stroke in China: a national serial cross-sectional study from 2003 to 2018. Stroke Vasc Neurol. 2023;8(3):238–48. 10.1136/svn-2022-001598 PMC10359805 36418056

[2] GBDS Collaborators (2021). Global, regional, and national burden of stroke and its risk factors, 1990–2019: a systematic analysis for the Global Burden of Disease Study 2019. Lancet Neurol.

[3] Gherghina ME, Peride I, Tiglis M, Neagu TP, Niculae A, Checherita IA (2022). Uric acid and oxidative stress-relationship with cardiovascular, metabolic, and renal impairment. Int J Mol Sci.

[4] Mandal AK, Mount DB (2015). The molecular physiology of uric acid homeostasis. Annu Rev Physiol.

[5] Feig DI, Kang DH, Johnson RJ (2008). Uric acid and cardiovascular risk. N Engl J Med.

[6] Kim JW, Byun MS, Yi D, Lee JH, Jeon SY, Ko K (2020). Serum uric acid, Alzheimer-related brain changes, and cognitive impairment. Front Aging Neurosci.

[7] Beydoun MA, Canas JA, Dore GA, Beydoun HA, Rostant OS, Fanelli-Kuczmarski MT (2016). Serum uric acid and its association with longitudinal cognitive change among urban adults. J Alzheimers Dis.

[8] Li J, Muraki I, Imano H, Cui R, Yamagishi K, Umesawa M (2020). Serum uric acid and risk of stroke and its types: the Circulatory Risk in Communities Study (CIRCS). Hypertens Res.

[9] Wang YF, Li JX, Sun XS, Lai R, Sheng WL (2018). High serum uric acid levels are a protective factor against unfavourable neurological functional outcome in patients with ischaemic stroke. J Int Med Res.

[10] Wu S, Pan Y, Zhang N, Jun WY, Wang C, Investigators for the Survey on Abnormal Glucose Regulation in Patients With Acute Stroke Across C (2017). Lower serum uric acid level strongly predict short-term poor functional outcome in acute stroke with normoglycaemia: a cohort study in China. BMC Neurol.

[11] Yang Y, Zhang Y, Li Y, Ding L, Sheng L, Xie Z (2018). U-shaped relationship between functional outcome and serum uric acid in ischemic stroke. Cell Physiol Biochem.

[12] Cheng D, Hu C, Du R, Qi H, Lin L, Wu X (2020). Serum uric acid and risk of incident diabetes in middle-aged and elderly Chinese adults: prospective cohort study. Front Med.

[13] Shao Y, Shao H, Sawhney MS, Shi L (2019). Serum uric acid as a risk factor of all-cause mortality and cardiovascular events among type 2 diabetes population: Meta-analysis of correlational evidence. J Diabetes Complications.

[14] Wang Y, Jing J, Meng X, Pan Y, Wang Y, Zhao X (2019). The Third China National Stroke Registry (CNSR-III) for patients with acute ischaemic stroke or transient ischaemic attack: design, rationale and baseline patient characteristics. Stroke Vasc Neurol.

[15] Kasner SE (2006). Clinical interpretation and use of stroke scales. Lancet Neurol.

[16] Gardener H, Romano LA, Smith EE, Campo-Bustillo I, Khan Y, Tai S (2022). Functional status at 30 and 90 days after mild ischaemic stroke. Stroke Vasc Neurol.

[17] Adams HP, Bendixen BH, Kappelle LJ, Biller J, Love BB, Gordon DL (1993). Classification of subtype of acute ischemic stroke. Definitions for use in a multicenter clinical trial. TOAST. Trial of Org 10172 in Acute Stroke Treatment. Stroke..

[18] Xu J, Cheng A, Song B, Zhao M, Xue J, Wang A (2022). Trimethylamine N-oxide and stroke recurrence depends on ischemic stroke subtypes. Stroke.

[19] Wang A, Tian X, Zuo Y, Chen S, Mo D, Zhang L (2022). Effect of changes in serum uric acid on the risk of stroke and its subtypes. Nutr Metab Cardiovasc Dis.

[20] Zheng S, Luo Y, Miao Q, Cheng Z, Liu Y, Lv K (2022). Serum uric acid levels and their changes and risk of stroke: a 7-year prospective cohort study in Northwest China. Cerebrovasc Dis.

[21] Su H, Liu T, Li Y, Fan Y, Wang B, Liu M (2021). Serum uric acid and its change with the risk of type 2 diabetes: a prospective study in China. Prim Care Diabetes.

[22] Wang L, Hu W, Miao D, Zhang Q, Wang C, Pan E (2017). Relationship between serum uric acid and ischemic stroke in a large type 2 diabetes population in China: a cross-sectional study. J Neurol Sci.

[23] Newman EJ, Rahman FS, Lees KR, Weir CJ, Walters MR (2006). Elevated serum urate concentration independently predicts poor outcome following stroke in patients with diabetes. Diabetes Metab Res Rev.

[24] Wang P, Li X, He C, Zhai Y, Sun H, Zhang Y (2019). Hyperuricemia and prognosis of acute ischemic stroke in diabetic patients. Neurol Res.

[25] Tang Y, Liang P, Chen J, Fu S, Liu B, Feng M (2018). The baseline levels and risk factors for high-sensitive C-reactive protein in Chinese healthy population. Immun Ageing.

[26] Lee KW, Shin D (2022). Concurrent presence of high serum uric acid and inflammation is associated with increased incidence of type 2 diabetes mellitus in Korean adult population. Sci Rep.

[27] Bai XC, Lu D, Bai J, Zheng H, Ke ZY, Li XM (2004). Oxidative stress inhibits osteoblastic differentiation of bone cells by ERK and NF-kappaB. Biochem Biophys Res Commun.

[28] Nabipour I, Sambrook PN, Blyth FM, Janu MR, Waite LM, Naganathan V (2011). Serum uric acid is associated with bone health in older men: a cross-sectional population-based study. J Bone Miner Res.

[29] Amaro S, Chamorro A (2011). Translational stroke research of the combination of thrombolysis and antioxidant therapy. Stroke.

[30] Chamorro A, Amaro S, Castellanos M, Segura T, Arenillas J, Marti-Fabregas J (2014). Safety and efficacy of uric acid in patients with acute stroke (URICO-ICTUS): a randomised, double-blind phase 2b/3 trial. Lancet Neurol.

[31] Amaro S, Llull L, Renu A, Laredo C, Perez B, Vila E (2015). Uric acid improves glucose-driven oxidative stress in human ischemic stroke. Ann Neurol.

[32] Wang J, Jin R, Wu Z, Liu Y, Jin X, Han Z (2022). Moderate increase of serum uric acid within a normal range is associated with improved cognitive function in a non-normotensive population: a nationally representative cohort study. Front Aging Neurosci.

[33] Yuan Z, Liu H, Zhou R, Gu S, Wu K, Huang Z (2023). Association of serum uric acid and fasting plasma glucose with cognitive function: a cross-sectional study. BMC Geriatr.

