# Correction: Moderate elevation of serum uric acid levels improves short-term functional outcomes of ischemic stroke in patients with type 2 diabetes mellitus

**DOI:** 10.1186/s12877-023-04242-0

**Published:** 2023-09-26

**Authors:** Yalun Dai, Yingyu Jiang, Luping Zhang, Xin Qiu, Hongqiu Gu, Yong Jiang, Xia Meng, Zixiao Li, Yongjun Wang

**Affiliations:** 1 Department of Neurology, Beijing Tiantan Hospital, Capital Medical University, Beijing, China; 2 China National Clinical Research Center for Neurological Diseases, Beijing Tiantan Hospital, Capital Medical University, No.119 South 4th Ring West Road, Fengtai District, Beijing, 100070 China; 3 Department of Obstetrics and Gynecology, Beijing Tiantan Hospital, Capital Medical University, Beijing, China; 4 Chinese Institute for Brain Research, Beijing, China

BMC Geriatrics (2023) 23:445

10.1186/s12877-023-04141-4

After publication of this article [1], the authors reported that in the Acknowledgements section the funding from the Ministry of Industry and Information Technology of the People’s Republic of China was omitted.

The original article [1] has been corrected.
