# Supplementary material for: Moderate elevation of serum uric acid levels improves short-term functional outcomes of ischemic stroke in patients with type 2 diabetes mellitus
Source: BMC Geriatr. 2023 Jul 19;23:445. doi: 10.1186/s12877-023-04141-4 (PMC10357838; doi:10.1186/s12877-023-04141-4)
Supplement: Supplementary file 1 — Additional file 1. Binary/multivariate logistic regression analyses of the association of $\Delta$ΔSUA with poor functional outcomes of IS in the population with HbA1c or FPG, table. [file 12877_2023_4141_MOESM1_ESM.docx]

**Additional file 1,** docx, Binary/multivariate logistic regression analyses of the association of $\Delta$SUA with poor functional outcomes of IS in the population with HbA1c or FPG, table

| $\boldsymbol{\Delta}$**SUA value (*μ*mol/L)** | **Population with HbA1c**^†^  **(N=873)** | | |  | **Population with FPG**^‡^  **(N=978)** | | |
| --- | --- | --- | --- | --- | --- | --- | --- |
|  | No. of event/No. at risk | OR (95% CI) | *P* value |  | No. of event/No. at risk | OR (95% CI) | *P* value |
| $\Delta$SUA$\leq$0 | 44/332 | reference | - |  | 53/373 | reference | - |
| $\Delta$SUA>0 | 50/541 | 0.54 (0.33-0.90) | **0.017** |  | 47/605 | 0.44 (0.27-0.72) | **0.001** |
| 0-50 | 22/259 | 0.55 (0.30-1.00) | **0.050** |  | 18/295 | 0.37 (0.20-0.70) | **0.002** |
| 50-100 | 12/172 | 0.34 (0.15-0.76) | **0.008** |  | 15/178 | 0.49 (0.24-1.01) | 0.052 |
| 100-150 | 10/64 | 1.24 (0.53-2.94) | 0.623 |  | 7/73 | 0.60 (0.23-1.58) | 0.304 |
| >150 | 6/46 | 0.50 (0.15-1.63) | 0.249 |  | 7/59 | 0.48 (0.17-1.39) | 0.177 |

†: adjusted for age, sex, BMI, NIHSS at admission, current smoking, heavy alcohol consumption, history of disease (i.e., heart disease, hypertension, and hyperlipidemia), CHOL, TG, LDL, and HbA1c

‡: adjusted for age, sex, BMI, NIHSS at admission, current smoking, heavy alcohol consumption, history of disease (i.e., heart disease, hypertension, and hyperlipidemia), CHOL, TG, LDL, and FPG

$\Delta$SUA, changes in serum uric acid; IS, ischemic stroke; OR, odds ratio; CI, confidence interval; BMI, body mass index; NIHSS, National Institutes of Health Stroke Scale; CHOL, total cholesterol; TG, triglyceride; LDL, low-density lipoprotein, HbA1c, hemoglobin A1c; FPG, fasting plasma glucose
