# Supplementary material for: Moderate elevation of serum uric acid levels improves short-term functional outcomes of ischemic stroke in patients with type 2 diabetes mellitus
Source: BMC Geriatr. 2023 Jul 19;23:445. doi: 10.1186/s12877-023-04141-4 (PMC10357838; doi:10.1186/s12877-023-04141-4)
Supplement: Supplementary file 2 — Additional file 2. Subgroup analysis of the association between $\Delta$ΔSUA and poor functional outcomes of IS, table. [file 12877_2023_4141_MOESM2_ESM.docx]

**Additional file 2,** docx, Subgroup analysis of the association between $\Delta$SUA and poor functional outcomes of IS, table

| $\boldsymbol{\Delta}$**SUA value (*μ*mol/L)** |  | **LAA** | |  |  | **CE** | |  |  | **SAO** | |  |  | **Others** | | ***P*_interatcion_** |
| --- | --- | --- | --- | --- | --- | --- | --- | --- | --- | --- | --- | --- | --- | --- | --- | --- |
|  | No. of event/No. at risk | OR (95% CI) | *P* value |  | No. of event/No. at risk | OR (95% CI) | *P* value |  | No. of event/No. at risk | OR (95% CI) | *P* value |  | No. of event/No. at risk | OR (95% CI) | *P* value |  |
| $\Delta$SUA$\leq$0 | 31/145 | reference | - |  | 0/14 | - | - |  | 8/130 | reference | - |  | 31/202 | reference | - |  |
| $\Delta$SUA>0 | 19/213 | 0.29 (0.13-0.63) | **0.002** |  | 7/34 | - | - |  | 6/185 | 0.37 (0.10-1.33) | 0.129 |  | 37/332 | 0.59 (0.32-1.07) | 0.081 | 0.768 |
| 0-50 | 6/92 | 0.24 (0.08-0.70) | **0.009** |  | 3/17 | - | - |  | 2/84 | 0.29 (0.05-1.64) | 0.161 |  | 16/166 | 0.49 (0.24-1.03) | 0.059 | 0.904 |
| 50-100 | 7/76 | 0.27 (0.08-0.85) | **0.025** |  | 2/11 | - | - |  | 1/55 | 0.31 (0.04-2.77) | 0.296 |  | 8/92 | 0.39 (0.14-1.08) | 0.070 |  |
| 100-150 | 3/29 | 0.29 (0.06-1.44) | 0.130 |  | 0/1 | - | - |  | 2/25 | 0.47 (0.04-5.47) | 0.546 |  | 9/42 | 1.88 (0.73-4.81) | 0.189 |  |
| >150 | 3/16 | 0.73 (0.14-3.86) | 0.708 |  | 2/5 | - | - |  | 1/21 | 0.87 (0.09-8.85) | 0.904 |  | 4/32 | 0.38 (0.09-1.61) | 0.189 |  |

$\Delta$SUA, changes in serum uric acid; IS, ischemic stroke; OR, odds ratio; CI, confidence interval; BMI, body mass index; NIHSS, National Institutes of Health Stroke Scale; CHOL, total cholesterol; TG, triglyceride; LDL, low-density lipoprotein; TOAST, Trial of ORG 10172 in Acute Stroke Treatment; LAA, large-artery atherosclerosis; CE, cardioembolism; SAO, small artery occlusion; Others, other determined or undetermined causes
