# Supplementary material for: Moderate elevation of serum uric acid levels improves short-term functional outcomes of ischemic stroke in patients with type 2 diabetes mellitus
Source: BMC Geriatr. 2023 Jul 19;23:445. doi: 10.1186/s12877-023-04141-4 (PMC10357838; doi:10.1186/s12877-023-04141-4)
Supplement: Supplementary file 3 — Additional file 3. Distribution of SUA levels in $\Delta$ΔSUA elevation subgroups, figure. [file 12877_2023_4141_MOESM3_ESM.docx]

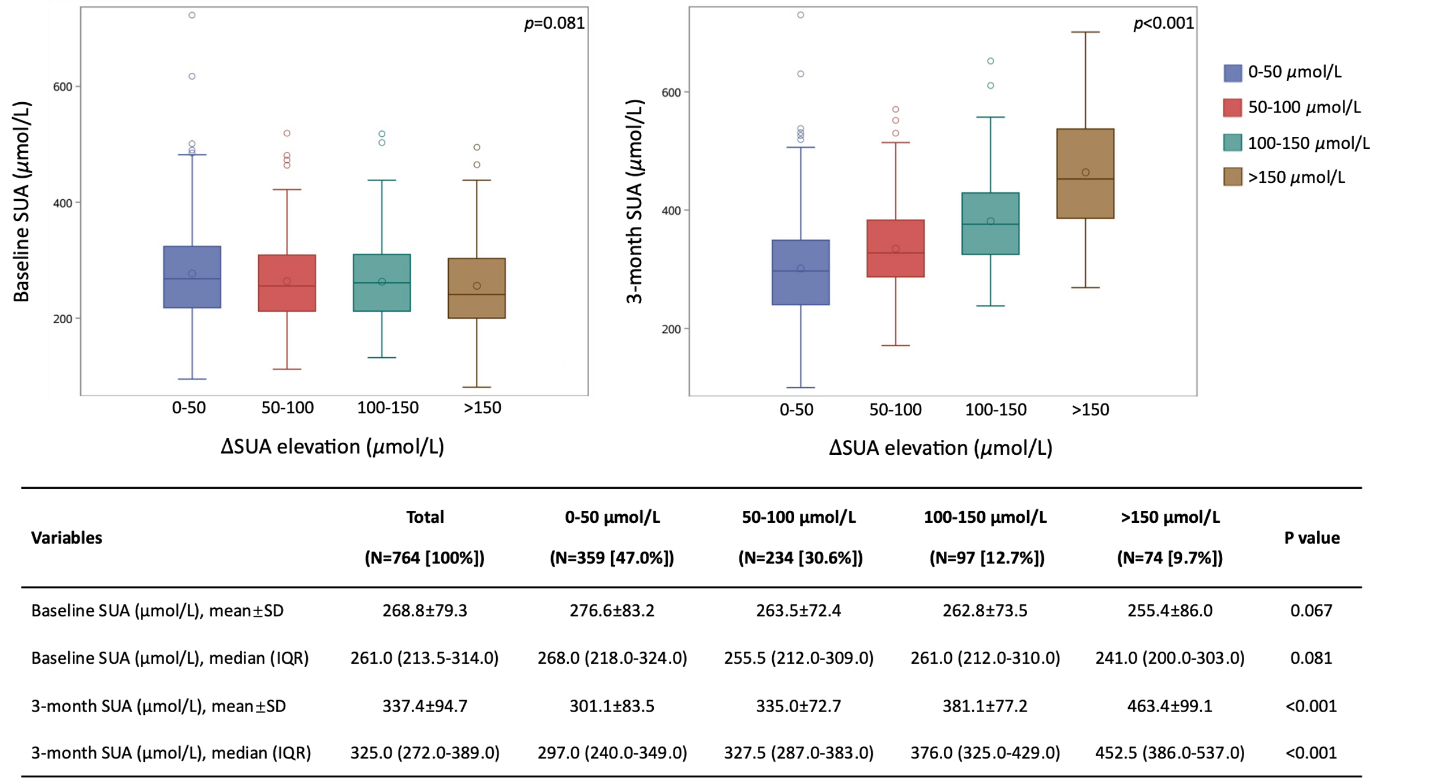
**Additional file 3,** docx, Distribution of SUA levels in $\Delta$SUA elevation subgroups, figure

SUA, serum uric acid; $\Delta$SUA, changes in serum uric acid; SD standard deviation; IQR, interquartile range
